# Efficient Generation of Bispecific Murine Antibodies for Pre-Clinical Investigations in Syngeneic Rodent Models

**DOI:** 10.1038/s41598-017-02823-9

**Published:** 2017-05-30

**Authors:** Aran F. Labrijn, Joyce I. Meesters, Matthew Bunce, Anthony A. Armstrong, Sandeep Somani, Tom C. Nesspor, Mark L. Chiu, Işil Altintaş, Sandra Verploegen, Janine Schuurman, Paul W. H. I. Parren

**Affiliations:** 1 Genmab, Utrecht, The Netherlands; 2 Biologics Research, Janssen Research and Development, LLC., Spring House, PA USA; 3 Discovery Sciences, Janssen Research and Development, LLC., Spring House, PA USA; 4 Department of Cancer and Inflammation Research, Institute of Molecular Medicine, University of Southern Denmark, Odense, Denmark; 5 Department of Immunohematology and Blood Transfusion, Leiden University Medical Center, Leiden, The Netherlands

## Abstract

Therapeutic concepts exploiting tumor-specific antibodies are often established in pre-clinical xenograft models using immuno-deficient mice. More complex therapeutic paradigms, however, warrant the use of immuno-competent mice, that more accurately capture the relevant biology that is being exploited. These models require the use of (surrogate) mouse or rat antibodies to enable optimal interactions with murine effector molecules. Immunogenicity is furthermore decreased, allowing longer-term treatment. We recently described controlled Fab-arm exchange (cFAE) as an easy-to-use method for the generation of therapeutic human IgG1 bispecific antibodies (bsAb). To facilitate the investigation of dual-targeting concepts in immuno-competent mice, we now applied and optimized our method for the generation of murine bsAbs. We show that the optimized combinations of matched point-mutations enabled efficient generation of murine bsAbs for all subclasses studied (mouse IgG1, IgG2a and IgG2b; rat IgG1, IgG2a, IgG2b, and IgG2c). The mutations did not adversely affect the inherent effector functions or pharmacokinetic properties of the corresponding subclasses. Thus, cFAE can be used to efficiently generate (surrogate) mouse or rat bsAbs for pre-clinical evaluation in immuno-competent rodents.

## Introduction

Preclinical efficacy of antibody therapeutics for oncology is commonly demonstrated using xenograft models in immuno-deficient mice, because these are less likely to elicit immune responses against either the xenograft or therapeutic antibody^1, 2^. However, to investigate increasingly more complex therapeutic concepts that go beyond targeting of the tumor or disease-tissue directly, the presence of relevant cross-talk between the intact immune system, disease associated immune cells, and disease tissue microenvironment are essential. As a consequence, the efficacy and safety of these complex concepts are often studied using syngeneic models in immuno-competent mice employing (surrogate) mouse antibodies to maximize the use of effector functions and avoid anti-drug responses^2–5^.

Efforts to improve therapeutic antibody efficacy include effector function enhancement through Fc-engineering^6^ and dual-targeting through the generation of bispecific antibodies (bsAbs)^7^. However, translating these functionally enhanced antibody formats into (surrogate) mouse formats for *in vivo* evaluation is not always straightforward as inter-species differences may compromise the functionality or the targeting strategy under investigation. In the case of bsAbs, early mouse surrogates were generated by fusing two hybridomas^8, 9^, thus generating heterogeneous mixtures of randomly paired heavy (H) and light (L) chains^10^, requiring extensive purification strategies to obtain the desired bsAb species (containing the relevant H_2_L_2_ assembly). Exploiting species-restricted HL pairing to reduce complexity in combination with differential Protein A elution (as observed when combining mouse IgG2a and rat IgG2b hybridomas) could significantly increase end-product yield^11, 12^. The rat sequences in these hybrid molecules, however, increase the potential for immunogenicity and may hamper correct engagement of the mouse effector functions, thus compromising proper translation of therapeutic concepts. Alternatively, tandem single chain variable fragments (scFvs)^13–15^ or chemically-crosslinked Fab fragments^16–19^ were used as surrogates. Their lack of Fc region, however, causes these fragment-based formats to have poor pharmacokinetic properties and disqualifies them for therapeutic concepts that require interaction with effector proteins. Surrogate formats with regular IgG architecture and based on a single murine subclass are expected to have the lowest potential immunogenicity and the most native functional characteristics.

**Figure 1:**
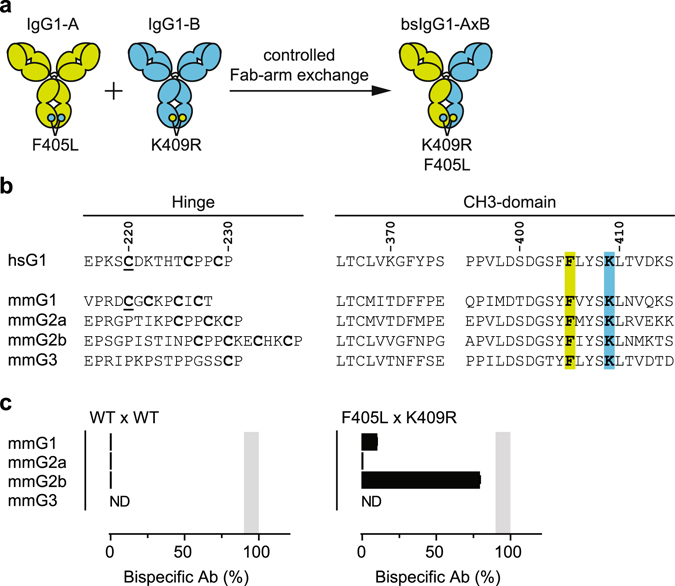
(**a**) Principle of controlled Fab-arm exchange (cFAE) for the generation of human IgG1-based bispecific antibodies. IgG1-A and IgG1-B molecules containing matching point mutations in their CH3 domains, F405L and K409R (EU numbering)^23^ respectively, are separately expressed and mixed in a 1 to 1 ratio under mild reducing conditions (5 h at 31 °C using 75 mM 2-MEA). This allows in the recombination of half-molecules driven by the matched mutations that enable dissociation of half-molecules in IgG1-A and IgG1-B and favor heterodimerization into IgG1-AB molecules. (**b**) Amino acid sequence alignment of human (*H*. *sapiens*; hs) and mouse (*M*. *musculus*; mm) hinge and CH3 regions. EU-numbering convention is used to annotate the amino acid residues. Cysteine residues in the hinge region involved in HC-HC linkage and LC-HC pairing (underlined) are indicated in bold. Residues F405 and K409 are indicated in green and blue, respectively. (**c**) Efficiency of cFAE as measured by HIC of mixtures of 2F8-derived and 7D8-derived mmIgG1, mmIgG2a or mmIgG2b parental antibodies, containing the indicated mutations (above panels). Data represent mean ± SEM of at least two separate experiments. ND = not done (due to limited sample availability). Shaded area represents 90–100% efficiency.

Controlled Fab-arm exchange (cFAE) was recently described as a versatile and robust method for the generation of human bsAb^20–22^ (Fig. 1a). cFAE involves mixing of two separately expressed parental antibodies, under controlled reducing conditions, to allow the recombination of antibody half-molecules (HL-pairs). The recombination is driven by two matching point-mutations, F405L and K409R (EU-numbering)^23^, one in each parental IgG, that weaken the non-covalent CH3-CH3 interaction in the parental Abs enough to allow for the dissociation of half-molecules, but at the same time, strongly favor the heterodimerization that promotes bsAb yield and post-exchange stability upon re-oxidation. To facilitate the investigation of dual-targeting strategies in immunocompetent mouse models, we now applied and optimized cFAE for the efficient generation of murine bsAbs.

## Results

### Application and optimization of controlled Fab-arm exchange for the efficient generation of murine bispecific antibodies

Although the reaction condition and matched point-mutations described for controlled Fab-arm exchange (cFAE) were optimized for the generation of human IgG1-based bispecific antibodies (bsAbs)^21, 22^, the approach can be used to generate bsAbs based on human IgG2, IgG3 and IgG4 subclasses^21^. To investigate whether cFAE could be applied to generate mouse bsAbs, the matched F405L and K409R mutations were introduced into chimeric antibodies containing the variable regions of human mAbs 2F8^24^ (human EGFR-specific) and 7D8^25^ (human CD20-specific), respectively, and mouse (*Mus musculus*, mm) IgG1, IgG2a, IgG2b or IgG3 constant regions. Subclass-matched mixtures of parental antibodies were subjected to cFAE and, without further purification, analyzed via hydrophobic interaction chromatography (HIC) to determine the efficiency of bispecific product formation^22^. Despite the sequence similarities in the relevant hinge and CH3 regions (Fig. 1b), the introduction of the matched F405L and K409R mutations only resulted in ~80% efficient cFAE in the mmIgG2b backbone (Fig. 1c). The mmIgG3-based constructs did not produce sufficient protein to assess cFAE and were not further investigated. The use of longer incubation times, higher concentrations of 2-mercaptoethylamine-HCl or stronger reducing agents, such as reduced glutathione, DTT, or Tris(2-carboxyethyl)phosphine, did not increase the efficiency of bsAb formation.

**Figure 2:**
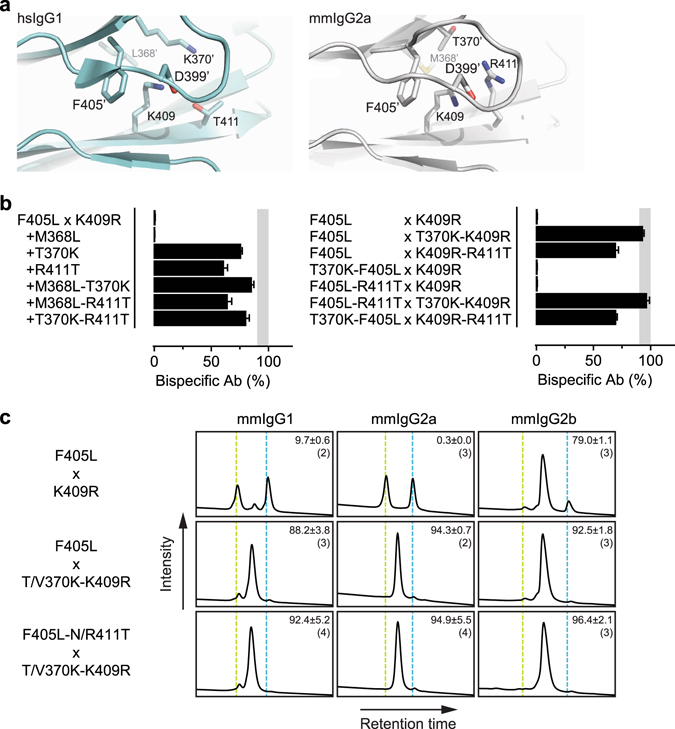
(**a**) Amino acid residues surrounding K409 at the CH3-CH3 interface of hsIgG1 (left panel) and mmIgG2a (right panel). Images were generated from PDB IDs 3AVE^54^ (hsIgG1) and 1IGT^61^ (mmIgG2a) using PyMOL software (Schrödinger). ‘Indicates residues on opposite CH3 domain. (**b**) Efficiency of cFAE as measured by HIC of symmetric (left panel) and asymmetric (right panel) mixtures of 2F8- and 7D8-derived chimeric parental antibodies. The chimeric parental antibodies contained human variable regions and human IgG1 constant regions, wherein only the CH3 domain was swapped with that of mouse IgG2a and containing the indicated point mutations. Data represent mean ± SEM of at least two separate experiments. Shaded area represents 90–100% efficiency. (**c**) Exemplary Hydrophobic Interaction Chromatography (HIC) profiles of bispecific antibodies generated by cFAE using combinations of 2F8- and 7D8-derived chimeric parental antibodies. The chimeric parental antibodies contained human variable regions and mouse constant regions that contained the indicated point mutations (above and left of panels, respectively). Numbers represent mean ± SEM (n) percentage of bispecific antibody product (middle peaks). Vertical lines correspond with the retention times of the individual 2F8 (green) and 7D8-derived (blue) parental antibodies.

The lack of recombination of mmIgG1- and mmIgG2a-based constructs could potentially be caused by both an incompatibility of the reaction conditions for efficient hinge reduction as well as an inability of the single point-mutations to enable dissociation and/or favor heterodimerization of the respective half-molecules. To exclude the potential of hinge incompatibility, human IgG1-based 2F8 and 7D8 constructs were generated wherein the CH3 domain was swapped for that of mmIgG2a, i.e. IgG1-CH3(mmG2a)-F405L and IgG1-CH3(mmG2a)-K409R respectively. In addition, these CH3-swapped constructs were used as model system to investigate the effect of additional CH3 domain mutations on cFAE efficiency. To guide the mutational analysis, the crystal structure of an intact mmIgG2a (PDB ID: 1IGT) was compared to that of human IgG1 Fc fragment (PDB ID: 3AVE) to identify residues which were spatially proximal to amino acid residues at positions 405 and 409 and different in sequence compared to human IgG1 (Fig. 2a). Thus amino acid residues T364, M368, T370, D371, E395 and R411 in mmIgG2a were identified (compared to S364, L368, K370, G371, P395 and T411 in human IgG1). Of these, amino acid residues at position 368, 370 and 411 were selected for introduction of mouse-to-human mutations, either alone or in combinations of two, into the IgG1-CH3(mmG2a)-K409R and IgG1-CH3(mmG2a)-F405L constructs. Mixtures of parental antibodies were subjected to cFAE and bispecific product formation was quantified by HIC.

The lack of recombination in the chimeric mmIgG2a-based constructs was recapitulated by the CH3-swapped IgG1-CH3(mmG2a)-K409R and IgG1-CH3(mmG2a)-F405L parental antibodies, thus excluding inefficient hinge reduction as the major cause. Addition of the M368L mutation in both parental antibodies had no effect, whereas the introduction of mutations T370K or R411T in both parental antibodies greatly increased the cFAE efficiency to 76 ± 2% and 61 ± 5%, respectively (Fig. 2b). Combining two of the three additional mutations in both parental antibodies did not cause any further significant increase in cFAE efficiency over the most effective single additional mutation (Fig. 2b). Surprisingly, the highest cFAE efficiencies (>90%) were obtained when the T370K mutation was introduced in the K409R-parental antibody only, i.e. IgG1-CH3(mmG2a)-T370K-K409R, and mixed with an IgG1-CH3(mmG2a)-F405L or IgG1-CH3(mmG2a)-F405L-R411T parental antibody (Fig. 2b).

To verify that the identified combinations also supported high cFAE efficiencies in chimeric constructs containing a complete mouse constant region, the mutations were introduced into mmIgG2a-based parental antibodies (i.e. mmIgG2a-T370K-K409R, mmIgG2a-F405L and mmIgG2a-F405L-R411T). In addition, to investigate whether the identified combinations were also applicable to other mouse subclasses, the equivalent mutations were introduced into parental antibodies based on mmIgG1 (i.e. mmIgG1-T370K-K409R, mmIgG1-F405L and mmIgG1-F405L-N411T) and mmIgG2b (i.e. mmIgG2b-V370K-K409R, mmIgG2b-F405L and mmIgG2b-F405L-N411T). Combinations of parental antibodies of the same subclass were subsequently mixed, subjected to cFAE and quantified by HIC. Indeed, the high cFAE efficiencies were observed for all tested mouse subclasses when the T/V370K-K409R parental antibodies were mixed with F405L or F405L-N/R411T parental antibodies of the same subclass (Fig. 2c). For all further studies F405L-N/R411T × T/V370K-K409R parental antibodies were used. Although cFAE is equally efficient in small volumes and/or at low protein concentrations, sample recovery can become less efficient under these conditions owing to general handling loss of the protein during removal of 2-MEA^22^. The mean protein recovery (mg protein recovered × 100/mg input protein) for the bench-scale bsAbs batches generated in this study was 75.2% (95% CI: 70.7–79.7%).

To assess whether the identified combinations of mutations were more generally applicable to other rodent IgG subclasses, the mutations were evaluated in rat (*Rattus norvegicus*, rn) IgG1, IgG2a, IgG2b and IgG2c backbones, which all led to similar results (Supplementary Fig. 1).

### T370K establishes human-like structural features at the mmIgG2a CH3-CH3 interface

**Figure 3:**
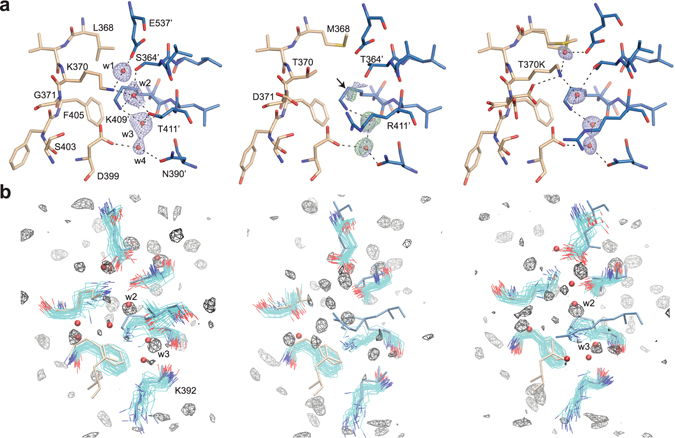
(**a**) Selected residues at the CH3-CH3 interface shown in stick for hsIgG1 (left, PDB ID: 3AVE)^54^, mmIgG2a (middle, PDB ID: 3ZO0^27^ from PDB_REDO databank^52^), and mmIgG2a-T370K mutant. Selected residues in hsIgG1 are labeled. Residues differing in sequence in mmIgG2a compared to hsIgG1 are also labelled. Structures are oriented similarly and opposing CH3 domains are indicated in tan and blue colors. Select water molecules are shown as red spheres (labeled) and hydrogen bonds (3.3 Å cut-off) as dashed lines. Water molecules depicted in mmIgG2a structure were not present in the PDB entry but were added in Coot and refined in real space consistent with available 2mFo-DFc density. 2mFo-DFc densities are shown at a contour of 1σ carved about water molecules at a distance of 1.5 Å (blue mesh). Additionally, the mFo-DFc density for mmIgG2a is shown at a contour of 3σ carved about placed water molecules at a distance of 1.5 Å (green mesh). An arrow points to 2mFo-DFc and mFo-DFc contoured as previously carved 1.5 Å about a pseudoatom positioned similarly to water w2 in PDB ID: 3AVE (not shown). mFo-DFc at this position was not visible at a contour of 3.5σ. (**b**) Side chain and water dynamics from MD simulations. Side chain conformations from the respective crystal structure are shown in licorice with residues D399, F405, T/K370 from one CH3 domain colored tan and E357′, S/T364′, K409′, T/R411′ and K392′ from the opposing CH3 domain colored blue. Water molecules from respective crystal structures are shown as red spheres. Fluctuations of the side chains in the MD simulation are indicated by overlaying 25 conformations sampled at an interval of 10 ns. For clarity, the fluctuations of the following residues are not shown: D399 for hsIgG1 (left), D399 and R411′ for mmIgG2a (middle), and D399 and R411′ for mmIgG2a T370K (right). Water occupancy maps contoured at an isolevel of 5 times bulk occupancy are shown in black wire mesh.

To determine the structural consequence of introducing the T370K mutation into the mmIgG2a CH3 domain, the crystal structure of the mmIgG2a-T370K Fc-region was determined. A network of conserved water molecules at the CH3-CH3 interface has previously been noted in high resolution crystal structures of the Fc-region of human IgG1 (Fig. 3a) and IgG2^26^. One water molecule, dubbed w2, is tetrahedrally coordinated by the side chains of T411, K409 and S364 from one CH3 domain and K370′ from the opposing CH3 domain, and the ability of R409 to displace this water, as observed in some human IgG4 CH3 domain-containing crystal structures, could contribute to the ability of IgG4 to undergo FAE^26^. No positionally equivalent water is present upon inspection of a 2.0 Å crystal structure of a wild-type mmIgG2a Fc-region (PDB ID: 3ZO0)^27^, and consistently, electron density for such a water molecule is weak (Fig. 3a). Interestingly, the T370K mutation was observed to restore a w2 equivalent water molecule at the mmIgG2a CH3-CH3 interface coordinated by K409 and T364 from one domain and T370K’ from the other (Fig. 3a). Furthermore, water occupancy maps and side chain fluctuations obtained from 250 ns room temperature molecular dynamics simulations were highly similar for human IgG1 and mmIgG2a-T370K at the CH3-CH3 interface around positions F405 and K409 but differed from that for wild-type mmIgG2a (Fig. 3b). In particular, simulations indicated the presence of w2 at the interface of human IgG1 and mmIgG2a-T370K but not at the interface of wild-type mmIgG2a, consistent with the observations from crystal structures. Additionally, fluctuations of water-coordinating residues remained close to the crystal structure for human IgG1 and mmIgG2a-T370K; however, a significant shift of the K409 side chain away from the crystal structure position was observed in the simulation of wild-type mmIgG2a. This movement of the K409 side chain coupled with a shorter side chain (Thr) at position 370 of the opposing domain altered the local water structure and lead to the absence of the w2 equivalent water molecule bridging the two CH3 domains.

### Regular functional properties are retained in cFAE-derived murine bispecific antibodies

As the matched mutations at the CH3-CH3 interface are located away from complement^28^ and Fcγ-receptor binding sites^29, 30^, effector functions were not expected to be impacted. To confirm this assumption, F405L, F405L-N/R411T and T/V370K-K409R variants of mmIgG1-7D8, mmIgG2a-7D8 and mmIgG2b-7D8 were compared to wild-type versions for their ability to induce complement-dependent cytotoxicity (CDC), antibody-dependent cellular cytotoxicity (ADCC), and antibody dependent cellular phagocytosis (ADCP). As controls, effector function-silenced variants of the mmIgG2a-derived parental antibodies were generated by introducing the L234A-L235A^29, 31^ mutations. The antibody variants were incubated with CD20-positive Raji cells and assessed for their ability to induce CDC in the presence of human serum as complement source and ADCC using a mouse FcγRIV ADCC Reporter Bioassay. Furthermore, antibody variants were incubated with CD20-positive Daudi cells and assessed for their ability to mediate phagocytosis by bone-marrow derived murine macrophages.

**Figure 4:**
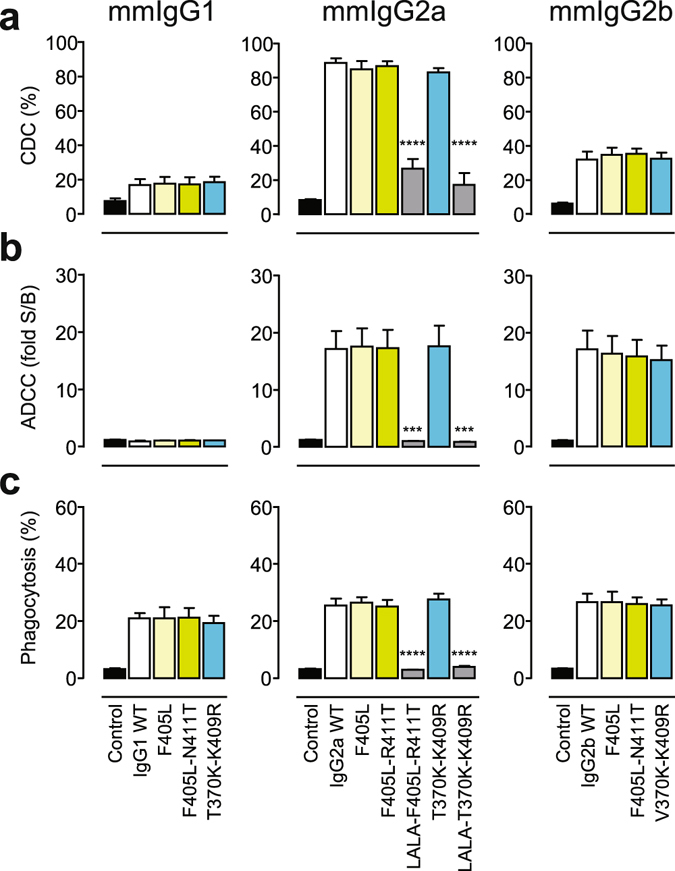
(**a**) CDC of Raji cells incubated with a fixed 10 μg/mL concentration of the indicated mmIgG1 (left), mmIgG2a (middle), or mmIgG2b (right) variants of mAb 7D8 in the presence of 20% (v/v) pooled human serum. (**b**) Surrogate ADCC activity of Jurkat-NFAT-mFcγRIV effector (E) cells induced by Raji target (T) cells incubated with a fixed 3 μg/mL concentration of the indicated variants (see A), with an E:T ratio of 1:1. (**c**) Phagocytosis of Daudi cells (T) by bone marrow-derived mouse macrophages (E) incubated with a fixed 1 μg/mL concentration of the indicated variants (see A), with an E:T ratio of 1:1. Data represent mean ± SEM of at least three experiments. Statistical significance (compared to WT) was determined by one-way ANOVA (***P *<* 0.001; ****P < 0.0001).

The analysis revealed no significant differences between the F405L, F405L-N/R411T, T/V370K-K409R, and wild-type 7D8 variants in the ability to induce CDC (Fig. 4a), (surrogate) ADCC (Fig. 4b), and phagocytosis (Fig. 4c). However, the introduction of the L234A-L235A (LALA) mutations in the mmIgG2a backbone completely abrogated the effector functions.

### Murine bispecific antibodies display regular pharmacokinetics

**Figure 5:**
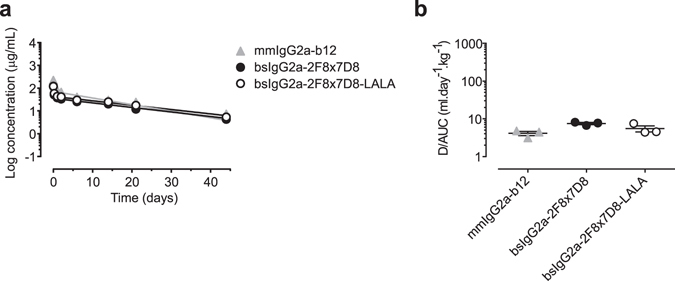
Total antibody plasma concentration over time (**a**) and plasma clearance rate (**b**) in C57Bl/6 J mice of mmIgG2a-derived bispecific antibodies. Mice (3 per group) were injected with the indicated antibodies (100 µg/mice). Blood samples were drawn at different times and plasma concentrations were determined by antigen-specific ELISA. Data represent mean ± SEM.

To verify that the matched mutations did not adversely affect the pharmacokinetic (PK) properties of the mouse Abs, a single dose (5 mg/kg) of bsIgG2a-2F8x7D8 was administered intravenously (i.v.) to C57Bl/6J mice and the antibody plasma concentration was followed over time. Simultaneously, an effector function-silenced bsAb variant, i.e. bsIgG2a-LALA, was evaluated and compared to wild-type Abs. The analysis showed comparable PK profiles and no significant differences in the clearance rates, indicating that introduction of the matching mutations, or the L234A-L235A mutations in mmIgG2a had no effect on the pharmacokinetic properties (Fig. 5). Furthermore, the normal linear profile of the elimination phase indicated that no anti-drug antibodies were elicited during the 44 day follow-up period, suggesting that the mutations were not immunogenic.

### Modeling dual-targeting concepts in syngeneic tumor model

**Figure 6:**
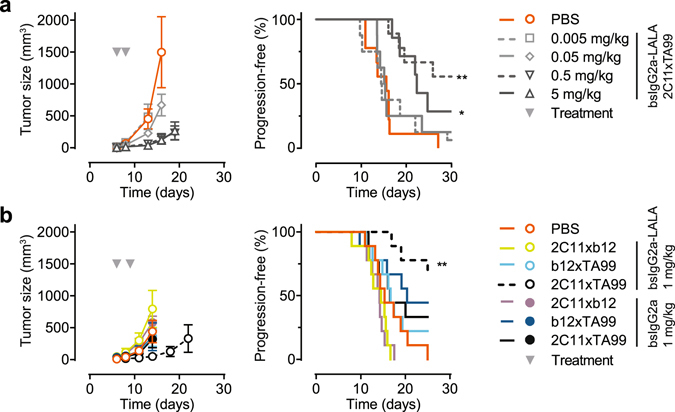
Evaluation of the *in vivo* efficacy of bsIgG2a-2C11xTA99(-LALA) in a syngeneic xenograft model with gp75-expressing B16-F10 tumor cells. (**a**) On day 6, mice were randomized (n = 7–9 per group) and treated intravenously with the indicated bsIgG2a-2C11xTA99-LALA doses, followed by a second dose at day 8 (arrowheads indicate treatment days). (**b**) On day 6, mice were randomized (n = 9 per group) and treated intravenously with the indicated bsAb variants at 1 mg/kg, followed by a second dose at day 9 (arrowheads indicate treatment days). Left panels show mean tumor volumes ± SEM (at time points where groups were still complete). Right panels show percentage of mice that remain progression-free (as defined by a tumor volume below 500 mm^3^). Statistical significance was determined by Mantel-Cox analysis (*P < 0.05; **P < 0.01).

One of the quintessential dual-targeting concepts championed by bsAbs is the redirection of T-cells to tumor cells^32^. To evaluate T-cell redirection *in vivo*, mouse bsAbs were generated by cFAE using anti-gp75 (TA99^33, 34^; tumor-specific arm) and anti-mouse CD3ε (2C11^35, 36^; T-cell-specific arm) parental antibodies. As controls, functionally monovalent bsAbs were also generated by substituting one of the parental antibodies with an irrelevant one (b12^37^; HIV-1gp120-specific arm). Subcutaneous B16-F10 tumors were established in C57Bl/6J mice and treated with two consecutive i.v. injections of bsIgG2a-2C11xTA99-LALA at days 6 and 8 post tumor inoculation. The antibodies were dosed at concentrations ranging from 0.005 mg/kg to 5 mg/kg and tumor-size was followed over time. Whereas low doses (0.005 and 0.05 mg/kg) of bsIgG2a-2C11xTA99-LALA displayed no significant anti-tumor activity, doses of 0.5 and 5 mg/kg could inhibit B16-F10 tumor growth significantly (Fig. 6a and Supplementary Fig. 2a). Equal doses of the functionally monovalent counterparts (2C11xb12 and b12xTA99) were unable to inhibit tumor growth (Supplementary Fig. 2b).

To evaluate the contribution of Fcγ-receptor interactions to the efficacy and toxicity of T-cell redirection, effector function competent (active) bsIgG2a variants of 2C11xTA99, 2C11xb12 and b12xTA99 were generated and compared to their effector function-silenced (inert) counterparts in the same model. At 1 mg/kg, both functionally monovalent active bsIgG2a variants (2C11xb12 and b12xTA99) showed no inhibition of B16-F10 tumor growth (Fig. 6b and Supplementary Fig. 3), similar to their corresponding inert bsIgG2a-LALA variants. In contrast to the inert equivalent, the active bsIgG2a-2C11xTA99 displayed no significant anti-tumor activity.

**Figure 7:**
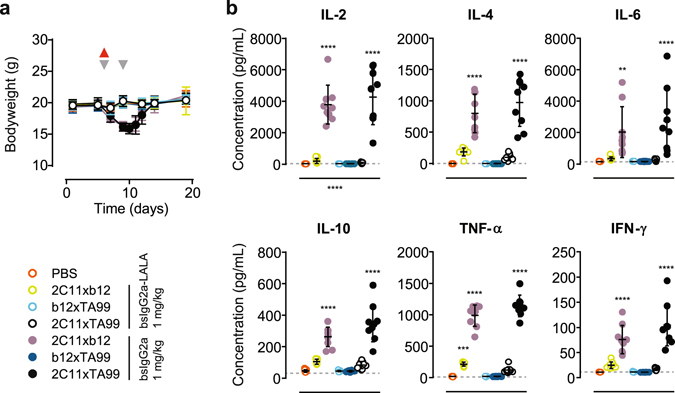
Evaluation of *in vivo* toxicity in a syngeneic B16-F10 xenograft model. Mice were dosed as described in Fig. 6B with the indicated bsAb variants. (**a**) Bodyweight followed over time (grey arrowheads indicate treatment days; red arrowhead indicates blood sampling). Data represent mean bodyweight ± SEM. (**b**) Cytokine concentrations at 1–2 h after the first dose. Data represent individual mice & mean ± SD. Statistical significance (compared to PBS control group) was determined by one-way ANOVA (**P < 0.01; ***P < 0.001; ****P < 0.0001).

Toxicity was only observed in the two active bsIgG2a variants targeting T-cells (2C11xTA99 and 2C11xb12) as revealed by the loss of bodyweight in these treatment groups (Fig. 7a). All mice had recovered by day 14 (8 days after first dose) and did not become worse after the second dose, indicating that the effects were transient and initiated by the first dose. Analysis of the serum, taken 1 h after injection of the first dose, revealed elevated levels of pro-inflammatory (IL-6, TNF-α), anti-inflammatory (IL-4, IL-10), and immuno-regulatory (IL-2, IL-4, IFN-γ) cytokines in these treatment groups (Fig. 7b), indicative of cytokine release syndrome^38^.

## Discussion

Much of the rationale for the successful clinical development of therapeutic antibodies targeting immune checkpoints has been obtained from syngeneic tumor models during their pre-clinical development^3, 39, 40^. Such use of animal models demonstrates the importance of translational modelling in immunocompetent mice containing an intact immune system to accurately capture the relevant biology being exploited, especially when targeting the tumor microenvironment rather than the tumor directly. The use of surrogate mouse antibodies in these models further enables proper engagement with murine effector functions and minimizes the development of anti-drug immune responses, thus adding to their transformational value. Likewise, surrogate mouse antibodies are critical for evaluation of chronic dosing in autoimmune disease models.

For the purpose of modeling dual-targeting concepts in immunocompetent mice, we applied and optimized controlled Fab-arm exchange (cFAE) for the efficient generation of surrogate murine bispecific antibodies (bsAbs). Using the previously described matched mutations, F405L and K409R^21^, in combination with additional N/R411T and T/V370K mutations respectively, bsAbs based on mouse IgG1, IgG2a and IgG2b backbones could be generated with high efficiency and yield (Supplementary Table 1), without compromising the intrinsic effector functions. The process has been scaled up to generate >1 g of murine bsAb without loss of efficiency and yield. Furthermore, in a bsIgG2a backbone the mutations did not affect the pharmacokinetic profile and were compatible with Fc-engineering mutations for silencing effector functions (i.e. L234A-L235A)^29, 31^. Likewise, targeting the same residues with similar mutations enabled the efficient generation of surrogate rat bsAbs by cFAE (Supplementary Fig. 1).

The mutational and structural analysis of the mouse CH3-CH3 interface suggested that the K409R mutation alone could not sufficiently destabilize the CH3-CH3 interaction to enable dissociation in the mmIgG1 and mmIgG2a backbones. In human IgG4, R409 is thought to destabilize the CH3-CH3 interface by causing an electrostatic clash with K370′ in the opposite CH3 domain^26, 41^. Parallel to observations with a K370T mutation in human IgG4, the T370 present in most mouse backbones hypothetically alleviates the electrostatic clash with R409′ in the opposite CH3 domain, thus enhancing CH3-CH3 interface stability^41, 42^. In human IgG1 (and IgG2), a conserved water network is observed, wherein one specific water molecule is thought to shield electrostatic repulsion between K370 and K409′^26^. A positionally equivalent water molecule was absent from the wild-type mmIgG2a CH3-CH3 interface. Introduction of the T370K mutation restored this water molecule, probably with an equivalent shielding function (Fig. 3). Similar to what has been observed with human IgG4^26^, the subsequent introduction of the K409R mutation could result in displacement of this water, thus enabling electrostatic repulsion and destabilization of the CH3-CH3 interface. The fact that combinations of equivalent mutations had similar effects in all the studied murine backbones despite additional sequence differences (Fig. 1b), suggested that this was a dominant factor governing CH3-CH3 interface stability.

Although alternative mouse bsAb formats with regular IgG architecture have been described, these either lacked^4^ or encompassed non-native effector-function interactions^12^. The surrogate mouse bsAbs produced by cFAE approach the native architecture of natural mouse IgG subclasses more closely, including their native effector function profiles. This allows modelling of the contribution of effector functions to both the preferred therapeutic activity as well as the unwanted side-effects. Furthermore, as cFAE uses two separately expressed parental antibodies in combination with post-production rearrangement of antigen-binding arms, there is no need for common light chains or species-restricted heavy-light chain pairing to evade end-product heterogeneity. Therefore, original pairings of heavy and light chain variable regions can be maintained, thus potentially increasing the exploitable repertoire of parental mAbs and their combinations to prepare diverse bsAbs^21, 43^.

The effector function-silenced (inert) surrogate mouse bsAbs described in this study were used to model murine T-cells redirection to B16-F10 tumor cells in a therapeutic setting. Despite the relative late dosing compared to other B16-F10-based T-cell redirection models^12, 13, 15, 17^, therapeutic activity was observed in the same dose-range reported for other surrogate bsAbs with regular IgG architecture^4, 12^. The use of an effector function competent (active) backbone resulted in transient T-cell-mediated toxicity, consistent with the occurrence of systemic cytokine release syndrome^38^. Although the active surrogate bsAbs could utilize additional Fc-mediated effector mechanisms (i.e. CDC, ADCC and ADCP), the lack of therapeutic efficacy of bsIgG2a-b12xTA99 *in vivo* suggests that Fc-mediated effector mechanisms are unable to control established tumors in this aggressive model at the tested doses. Furthermore, the therapeutic difference between the inert (bsIgG2a-2C11xTA99-LALA) and active (bsIgG2a-2C11xTA99) bsAbs implies that the Fc-mediated effector mechanisms hamper T-cell redirection, probably by canceling the conditional activation by the tumor target and systemically depleting or exhausting T-cells through the CD3 arm. Although further in-depth evaluation is essential for a full understanding, the combined results suggest that T-cell redirection represents a very potent mode-of-action by itself and should not be combined with regular Fc-mediated effector functions in order to minimize toxicity and maximize efficacy.

Besides proper interaction between tumor cells and a fully competent immune system, there are other advantages of working with syngeneic xenograft models compared to human xenografts in SCID mice, humanized mice or genetically engineered mouse models. Immunocompetent mice are readily accessible, relatively cheap and mouse xenografts are easily established, although the number of suitable mouse cell-lines for grafting is limited^1, 44, 45^. This repertoire, however, can be greatly expanded by transfecting the tumor target of interest into the available mouse cell-lines^4, 12–15, 17^. The differences in tumorigenesis between mouse and human tumors, e.g. the lower level of genetic complexity and the lack of a chronic inflammatory environment due to rapid growth of murine tumors, represent important caveats in syngeneic cancer modelling^1, 44, 45^. Thus, syngeneic modeling should not be used as the exclusive validation of any preclinical concept, but rather as an integrated part of an *in vivo* validation strategy.

Here we show that surrogate murine bsAbs with native antibody architecture and functionality can be easily generated through cFAE and represent valuable tools for syngeneic modeling of therapeutic dual-targeting concepts in immunocompetent rodents. Although we describe an example in the field of oncology, the surrogate murine bsAbs can be applied to other disease models employing immunocompetent mice. Differences in binding-profiles of murine IgG subclasses to Fcγ-receptors and complement^46, 47^, may preclude direct translation of Fc-engineering efforts aimed at enhancing effector functions in humans^48^. Further expansion of the repertoire of murine surrogates to include such effector function enhanced formats would broaden the range of therapeutic concepts that could be addressed with syngeneic modelling in rodents for future translational research.

## Methods

### Cell lines

FreeStyle^TM^ 293F (HEK-293F), Expi293F^TM^ cells (Expi) and FreeStyle^TM^ CHO-S (CHO-S) cells were cultured in FreeStyle^TM^ 293 expression medium and FreeStyle^TM^ CHO expression medium, respectively (Invitrogen). Additional cell lines were obtained from the American Type Culture Collection (ATCC). B16-F10 (C57Bl/6-derived mouse skin melanoma) cells were cultured in DMEM medium containing Ultraglutamin 1 (Lonza), supplemented with 10% (v/v) Donor Bovine Serum with Iron (Life Technologies) and Pen/Strep (Lonza). Raji (human CD20-positive Burkitt’s lymphoma) cells and Daudi (human CD20-positive Burkitt’s lymphoma) cells were cultured in RPMI 1640 medium (Lonza), supplemented with 10% (v/v) Donor Bovine Serum with Iron, 1% (w/v) L-glutamine 200 mM in 0.85% (w/v) NaCl solution (Lonza), 1% Sodium Pyruvate (Lonza) and Pen/Strep (Lonza). All cell lines were maintained at 37 °C in a 5% CO_2_ humidified incubator.

### Cloning and production of antibodies

Antibody heavy-chain expression vectors were constructed by *de novo* synthesis (Geneart) of codon optimized VH coding regions of human mAbs 7D8 (human CD20-specific)^25^, 2F8 (human EGFR-specific)^24^, b12 (HIV-1 gp120-specific)^37^, Armenian hamster mAb 2C11 (mouse CD3-specific)^35, 36^, or murine mAb TA99 (human and mouse TYRP1/gp75-specific)^33, 34^, genetically fused to the heavy-chain constant coding regions of mouse (mm)IgG1 (IMGT Accession number J00453), mmIgG2a (V00825), mmIgG2b (V00763) or mmIgG3 (X00915) and inserted into expression vector pcDNA3.3 (Invitrogen). Alternatively, human IgG1 (Y14737) expression vectors of mAbs 7D8 and 2F8 were constructed containing mmIgG2a CH3 domains, i.e. IgG1-CH3(mmG2a). Likewise, separate light-chain expression vectors were constructed by inserting the appropriate VL coding regions in frame with the CL coding regions of the mouse (V00807) or human (J00241) kappa light chain into expression vector pcDNA3.3. A QuikChange site-directed mutagenesis kit (Stratagene) was used to introduce the L234A, L235A, D265A, F405L, K409R, V370K, T370K, N411T and R411T, (EU numbering conventions are used throughout the manuscript) point-mutations.

All antibodies were produced under serum-free conditions by co-transfecting relevant heavy and light chain expression vectors in FreeStyle^TM^ 293-F or Expi293F^TM^ cells, using 293fectin^TM^ or ExpiFectamine^TM^ 293, respectively (LifeTechnologies), according to the manufacturer’s instructions.

Antibodies were purified by protein A affinity chromatography (MabSelect SuRe; GE Health Care), dialyzed overnight to PBS, and filter-sterilized over 0.2-µM filters. Alternatively, antibodies were purified by protein G affinity chromatography (GE Health Care). The purity was determined by SDS-PAGE/CE-SDS and the concentration was measured by absorbance at 280 nm (specific extinction coefficients were calculated for each protein). Batches of purified antibody were tested by high-performance size-exclusion chromatography (HP-SEC) to determine the presence of aggregates or degradation products. Purified antibodies were stored at 2–8 °C. Endotoxin levels of batches used *in vivo* were below 0.1 endotoxin units/mg IgG.

### Controlled Fab-arm exchange

Equimolar amounts of relevant IgG1-F405L and IgG1-K409R antibodies were mixed and incubated with 2-Mercaptoethylamine (2-MEA; Sigma) at a final concentration of 1 mg/mL per antibody. The final concentration of 2-MEA was 75 mM. The mixtures were typically incubated for 5 h at 31 °C. To remove 2-MEA, the mixtures were buffer-exchanged against PBS using PD-10 desalting columns (5 kDa molecular weight cut-off; GE Healthcare) or dialysis using Slide-A-Lyzer cassettes (10 kDa molecular weight cut-off; Pierce). Samples were stored overnight at 4 °C to allow for the re-oxidation of the disulfide bonds.

### Hydrophobic interaction chromatography (HIC)

The efficacy of cFAE was assessed by hydrophobic interaction chromatography (HIC). For this, samples of the parental antibodies and the bispecific antibody product, generated by cFAE, were diluted twofold with HIC mobile phase buffer A (15.4 mM K_2_HPO_4_, 9.6 mM KH_2_PO_4_, 1,5 M (NH_4_)_2_SO_4_; pH 7.0) to a final concentration of 0.25 mg/mL for injection into the HPLC. The IgG molecules with different hydrophobic properties were separated using a Butyl-NPR, 2.5 μm, 4.6 × 35 mm HIC-HPLC column (Tosoh Bioscience) with a flow rate of 1 mL/min. 50 µL was injected and elution was performed with a 12-min gradient of HIC mobile phase buffer A to HIC mobile phase buffer B (15.4 mM K_2_HPO_4_, 9.6 mM KH_2_PO_4_; pH 7.0) and detection occurred at 280 nm. Empower 3 software (Waters) was used to assign and integrate peak areas. Chromatograms of the parental antibodies were used as reference to identify their position in the end-product. The relative peak areas of the bispecific antibody and residual parental antibodies were used to calculate the efficiency of the cFAE reaction.

### Protein Crystallography

Mouse IgG2a-T370K Fc mutant was recombinantly expressed in HEK293 cells and purified by Protein A affinity chromatography followed by size exclusion chromatography at Sino Biological, Inc. (China). Crystallization experiments employing the sitting drop vapor diffusion method were set up as 300 nL drops in Corning 3550 96-well plates. Crystals were grown at 20 °C. Initial crystals obtained in 23–25% (w/v) PEG 1,000, 0.1 M HEPES, pH 7.5, 5% (w/v) PEG 400 were used as seeds in subsequent refinement of crystallization conditions. Final crystals were obtained with a reservoir solution comprising 40% (w/v) PEG 200, 0.1 M MES, pH 6.5.

Crystals were cryoprotected in reservoir solution supplemented with 20% (v/v) glycerol, and snap frozen in LN_2_. X-ray diffraction data were collected using a 1.000 Å wavelength at the Advanced Photon Source (APS) beamline 17-ID (IMCA-CAT) at Argonne National Laboratory equipped with a DECTRIS Pilatus 6 M pixel array detector and were processed with the program XDS^49^.

Initial phases were determined by the method of molecular replacement using as a search model isolated CH2 and CH3 domains from previously refined internal structure of similar composition. The initial model underwent rounds of rebuilding and refinement using the programs Coot^50^ and PHENIX^51^, respectively. Toward the end of refinement the structure was processed with PDB_REDO^52^, and final refinement was performed using the program Refmac5^53^. The final model had good stereochemical quality with 98.4% percent of residues in the favorable region of the Ramachandran map and without Ramachandran outliers. Data processing and refinement statistics are presented in Supplementary Table 2.

### Molecular Dynamics Simulation

Explicit solvent molecular dynamics (MD) simulations of the CH3 dimer were performed for human IgG1 wild-type (PDB ID: 3AVE)^54^, mmIgG2a wild-type (PDB ID: 3ZO0)^27^, and the crystal structure of mmIgG2a-T370K determined here. For each system, the crystal structures of the CH3 dimer were used as the starting conformation. The simulations were performed under NPT conditions with a fixed number of atoms, constant pressure of 1 bar, and constant temperature of 300 K. The simulations were set up in Maestro and run using the GPU version of Desmond^55^, both part of the Schrodinger 2016-3 suite (Schrodinger, LLC, New York, NY). In the initial setup, the crystal structures were solvated in a cubic box of size 70 Å. Due to the same simulation box size and similar size and shape of the different CH3 domains, all simulations had roughly the same number of water molecules (~10,000). The OPLS3^56^ force field was used for the protein and the SPC^57^ model was used for the water molecules. Initial equilibration was performed using standard protocol (see, for example, ref. 58) in Desmond. The production run was 250 ns long using a time step of 2 fs and conformations were saved every 100 ps generating a trajectory with 2500 frames. The frames were aligned on the respective crystal structures based on backbone atoms of residues 370, 371, 399 and 405 from one CH3 domain and residues 364′ and 409′ from the opposite CH3 domain. In order to inspect the water structure in the interface, a three dimensional normalized histogram of the water oxygen atoms was computed after discretizing the simulation box into cubic cells of side 0.5 Å. The water occupancy analysis was performed using the grid utility in the AmberTools 16^59^ and visualized in VMD^60^.

### Complement dependent cytotoxicity (CDC)

The capacity to induce CDC was assessed essentially as described^25^. Briefly, target cells (1 × 10^5^ cells) were pre-incubated at 21 °C for 15 min with serial diluted antibodies. Pooled human serum (20% (v/v)) was added as a source of complement and cells were incubated at 37 °C for an additional 45 min. Cells were then put on ice and viability was determined by staining with propidium iodide (PI) and detected using a FACSCanto II flow cytometer (BD Biosciences). CDC activity was expressed as percentage of lysis as determined from the percentage of PI-positive cells.

### Surrogate antibody dependent cell-mediated cytotoxicity (ADCC) reporter bioassay

The capacity to induce ADCC was assessed by ADCC reporter Bioassay (Promega). Briefly, Raji target (T) cells (7.5 × 10^4^ cells), serial diluted antibodies and freshly thawed Jurkat-NFAT-mFcγRIV effector (E) cells were incubated together (in an E:T ratio of 1:1) in 75 μL/well of ADCC assay buffer in a 96-well white OptiPlate (Perkin Elmer) for 6 h at 37 °C. Next, 75 μL/well of Bio-Glo luciferase reagent was added according to the manufacturer’s instructions and incubated 15 min at RT. Luminescence was recorded in relative light units (RLU) on an EnVision^®^ Multilabel Reader (Perkin Elmer). ADCC activity was expressed as fold induction and was calculated using the following formula: fold = S/B, where S = signal (experimental RLU–background RLU) and B = background (control [no antibody] RLU–background RLU).

### Animals

Female C57Bl/6 J mice and BALB/c mice were obtained from Charles River Laboratories. The animals were housed and handled in accordance with good animal practice as defined by FELASA, in an AAALAC and ISO 9001:2000 accredited animal facility (GDL). The mice were kept in IVC cages with water and food provided ad libitum. Ear tags were used for mouse identification. Mice participating in experiments were checked daily for signs of toxicity and discomfort. All animal experiments were performed in compliance with the Dutch animal protection law (WoD) translated from the directives (2010/63/EU) and if applicable, the Code of Practice “animal experiments for cancer research” (Inspection V&W, Zutphen, The Netherlands, 1999). All animal experiments were approved by the Ethical committee of Utrecht.

### Culturing bone marrow-derived macrophages

Bone marrow-derived macrophages were obtained by flushing the femoral and tibial bones from the hind legs of C57Bl/6-derived human FcRn transgene mice [B6.Cg-Fcgrt^tm1Dcr^ Tg(FCGRT)32Dcr/DcrJ] (15–17 week old) with bone marrow medium (high glucose DMEM, without L-glutamine, supplemented with 50 IU/mL penicillin, 50 μg/mL streptomycin, 10% (v/v) heat-inactivated donor bovine serum and 2 mM L-glutamine) until the bones turned white. Cells were passed through a cell strainer and seeded in petri dishes at a cell concentration of 1.25 × 10^5^ cells/mL in 10 mL. Cells were cultured for 7–8 days at 37 °C, 5% CO_2_ in bone marrow medium supplemented with 50 U/mL M-CSF (PeproTech Inc.). After 3–4 days of incubation, 5 mL/dish fresh bone marrow medium with M-CSF was added.

### Antibody-dependent Cellular Phagocytosis (ADCP)

Bone marrow derived macrophages were washed with PBS and harvested by incubating the cells for 10 min at 37 °C with 2 mL Versene (Gibco). Detached cells were collected in a 50 mL tube and Versene was inactivated by adding bone marrow medium. Cells were washed twice and re-suspended in 5 mL ADCP working medium (high glucose DMEM, without L-glutamine and phenol red, supplemented with 50 IU/mL penicillin, 50 μg/mL streptomycin, 10% (v/v) heat-inactivated donor bovine serum, 2 mM L-glutamine and 25 mM HEPES). Cells were counted and the concentration adjusted to 0.5 × 10^6^ cells/mL with ADCP working medium. The bone-marrow derived macrophages were seeded into 96-well culture plates (200 µL/well) and allowed to adhere overnight at 37 °C, 5% CO_2_.

The following day, Daudi cells were harvested and labeled for 25 min at 37 °C with 0.01 nM Calcein-AM (Molecular probes), according to the manufacturer’s instructions. Labeled Daudi cells were washed twice with ADCP working medium, counted and the concentration adjusted to 1 × 10^6^ cells/mL. Supernatant from the bone-marrow derived macrophages culture plates was removed and Daudi target cells (T) were added to the macrophage effector cells (E) at an E:T ratio of 1:1 in the presence of a fixed antibody concentration of 1 μg/mL. After incubating 4 h at 37 °C, 5% CO_2_, cells were washed once with PBS and macrophages detached with Trypsin-EDTA (Gibco), Supernatant (containing target cells), washing step and macrophages were all collected in a 96-well U-bottom plate. Samples were stained with F4/80-PE (AbD Serotec) and CD19-APC (DAKO). ADCP was evaluated on a FACSCanto II flow cytometer (BD Biosciences) and defined as percentage of macrophages that had phagocytized. Percentage of phagocytosis was calculated using the following gate settings: the percentage of Calcein-AM-positive and CD19-negative cells within the F4/80-positive cells.

### Pharmacokinetic (PK) analysis

Antibodies (100 µg per mouse) were administered intravenously (i.v.) to groups (n = 3) of C57Bl/6 J mice and blood samples were drawn from the saphenous vein at 10 min, 3 h, 1, 2, 7, 14, 21 and 44 days after administration. Blood was collected in heparin-containing vials, which were kept on ice, and centrifuged (5 minutes at 10,000 g) to separate the plasma from cells. Plasma was transferred to a new vial and stored at −20 °C for determination of antibody levels.

### Quantitative IgG ELISA

Antibody plasma concentrations were determined using indirect antigen capture ELISAs. In short, ELISA plates were coated overnight with 2 μg/mL of EGFR-ECD-His (Genmab) in PBS at 4 °C. Alternatively, ELISA plates were coated overnight with 0.5 μg/mL of gp120-JRFL (Progenics) in PBS at 4 °C. The plates were subsequently washed and blocked with PBS-B (PBS/0.2% (w/v) BSA) for 1 h at 20 °C. Next, the plates were washed and incubated with diluted plasma samples in PBS-TB (PBS/0.05% (v/v) Tween-20/0.2% (w/v) BSA) for 1 h at 20 °C under shaking conditions (300 rpm). Bound antibodies were detected by HRP-labeled goat anti-mouse IgG (Jackson ImmunoResearch) and ABTS substrate (Roche Diagnostics). The color development reaction was stopped by addition of an equal volume of oxalic acid and absorbance was measured at 405 nm. IgG was quantified by nonlinear regression curve-fitting (GraphPad Software) using the injection mixtures as reference.

### *In vivo* efficacy

B16-F10 cells, cultured to 70% confluency, were harvested and injected subcutaneously (1 × 10^5^ cells in PBS) into the right flank of female C57Bl/6J mice (8–11 weeks old). At day six post tumor inoculation, mice were randomized (n = 9 per group unless indicated otherwise) and treated intravenously with bispecifc antibodies followed by a second treatment at day 9. Tumor volumes were measured twice a week and calculated from digital caliper measurements as 0.52 × length × width^2^ (in mm^3^). Animals were sacrificed when tumor volumes exceeded 1500 mm^3^ or when serious clinical signs were observed.

### Cytokine Quantification

Plasma samples, collected 1–2 h after treatment, from tumor bearing mice, were assayed for cytokine levels using a V-PLEX Proinflammatory Panel 1 (mouse) Kit (MSD technologies), according to manufacturer’s instructions. Briefly, 50 μL/well of calibrator, sample and negative control (normal mouse serum) dilutions were added to the MSD multiplex plates and incubated for 2 h at RT under shaking conditions. After washing of the plates, 25 μL/well detection Ab dilutions was added and incubated for another 2 h at RT under shaking conditions. Subsequently plates were washed and 150 μL/well 2x Read buffer T (MSD technologies) was added. Chemiluminescence was read on a MSD Sector Imager 6000 and cytokine concentrations were quantified by nonlinear regression curve-fitting (GraphPad Software) using the calibrator samples as reference.

### Statistical analysis

Data sets were compared using one-way ANOVA analysis (Dunnett’s multiple comparison) (GraphPad Prism for Windows, version 5.01; GraphPad). Log-rank Mantel-Cox analysis was applied to tumor progression curves (SPSS statistics 20; IBM). Statistical significance was accepted when P < 0.05.

## Electronic supplementary material

Labrijn et al. Supplementary Info
