# Supplementary material for: Efficient Generation of Bispecific Murine Antibodies for Pre-Clinical Investigations in Syngeneic Rodent Models
Source: Sci Rep. 2017 May 30;7:2476. doi: 10.1038/s41598-017-02823-9 (PMC5449386; doi:10.1038/s41598-017-02823-9)
Supplement: Supplementary file 1 — Labrijn et al. Supplementary Info [file 41598_2017_2823_MOESM1_ESM.pdf]

Supplementary Information

EFFICIENT GENERATION OF BISPECIFIC MURINE ANTIBODIES FOR PRE-CLINICAL INVESTIGATIONS IN SYNGENEIC RODENT MODELS

Aran F. Labrijn[1], Joyce I. Meesters[1], Matthew Bunce[2], Anthony A. Armstrong[2], Sandeep Somani[3], Tom C. Nesspor[2], Mark L. Chiu[2], Işil Altintaş[1], Sandra Verploegen[1], Janine Schuurman[1], Paul W.H.I. Parren[1,4,5*]

[1]Genmab, Utrecht, The Netherlands; [2]Biologics Research, Janssen Research and Development, LLC., Spring House, PA; [3]Discovery Sciences, Janssen Research and Development, LLC., Spring House, PA; [4]Department of Cancer and Inflammation Research, Institute of Molecular Medicine, University of Southern Denmark, Odense, Denmark, [5]Department of Immunohematology and Blood Transfusion, Leiden University Medical Center, Leiden, the Netherlands

*Corresponding author: p.parren@genmab.com

SUPPLEMENTARY METHODS

Cloning and production of antibodies. Antibody heavy-chain expression vectors were constructed by *de novo* synthesis (Geneart) of codon optimized VH coding regions of human mAbs 7D8 (human CD20-specific[1]) and 2F8 (human EGFR-specific[2]), genetically fused to the heavy-chain constant coding regions of rat (rn)IgG1 (IMGT Accession number AABR03048905), rnIgG2a (AABR03049560), rnIgG2b (AABR03048905) or rnIgG2c (AABR03049912) and inserted into expression vector pcDNA3.3 (Invitrogen). Likewise, separate light-chain expression vectors were constructed by inserting the appropriate VL coding regions in frame with the CL coding regions of the rat (V01241) kappa light chain into expression vector pcDNA3.3. A QuikChange site-directed mutagenesis kit (Stratagene) was used to introduce the F405L, K409R, S370K, T370K, N411T and S411T point-mutations.

All antibodies were produced under serum-free conditions by co-transfecting relevant heavy and light chain expression vectors in FreeStyle™ 293-F or Expi293F™ cells, using 293fectin™ or ExpiFectamine™ 293, respectively (LifeTechnologies), according to the manufacturer's instructions.

The antibodies were purified by protein G affinity chromatography (GE Health Care). The purity was determined by SDS-PAGE/CE-SDS and the concentration was measured by absorbance at 280 nm (specific extinction coefficients were calculated for each protein). Batches of purified antibody were tested by high-performance size-exclusion chromatography (HP-SEC) for

aggregates or degradation products. Purified antibodies were stored at 2-8℃. Endotoxin levels of batches used *in vivo* were below 0.1 endotoxin units/mg IgG.

Controlled Fab-arm exchange (cFAE) and quantification of cFAE efficiency by HIC were performed as described in the main text.

***In vivo* efficacy.** B16-F10 cells, cultured to 70% confluency, were harvested and injected subcutaneously ($1x10^5$ cells in PBS) into the right flank of female C57Bl/6J mice (8-11 weeks old). At day six post tumor inoculation, mice were randomized (n=9 per group) and treated intravenously with bispecific antibodies followed by a second treatment at day 8 or 9. Tumor volumes were measured twice a week and calculated from digital caliper measurements as 0.52 x length x width^2 (in mm^3). Animals were sacrificed when tumor volumes exceeded 1500 mm^3 or when serious clinical signs were observed.

Supplementary Table 1: cFAE efficiencies of mouse bsAbs batches

	2F8x7D8	2C11xTA99	b12xTA99	2C11xb12
bsIgG1	92.4 ± 5.2 (4)*			
bsIgG2a	95.7 ± 5.1 (5)	93.2 (1)	97.1 (1)	93.71 (1)
bsIgG2a-L234A-L235A	98.8 (1)	94.6 ± 3.3 (5)	93.2 ± 0.8 (2)	95.9 ± 0.2 (2)
bsIgG2b	96.4 ± 2.1 (3)			

*mean % ± SD (n),

Mean protein recovery (mg recovered x 100/ mg input) was 75.2% (95% CI: 70.7-79.7)

Supplementary Table 2 Data collection and refinement statistics

**Data collection**	
Space group	$P2_1$
Cell dimensions	
a,b,c (Å)	51.22, 86.35, 67.56
α, β, γ (°)	90.00, 111.58, 90.00
Resolution (Å)	50.00-1.55 (1.59-1.55)*
R_{sym}	0.040 (0.597)
<I / σI>	15.47 (2.41)
Completeness (%)	98.3 (98.6)
Redundancy	3.3 (3.4)
**Refinement**	
Resolution (Å)	47.64-1.55
No. reflections	74,111
R_{work} / R_{free}	0.169 / 0.195
No. atoms	
Protein	3,418
Carbohydrate	220
Solvent	383
B-factors	
Protein	27.88
Carbohydrate	41.45
Solvent	41.22
R.m.s deviations	
Bond lengths (Å)	0.014
Bond angles (°)	1.776

* Highest resolution shell is shown in parenthesis.

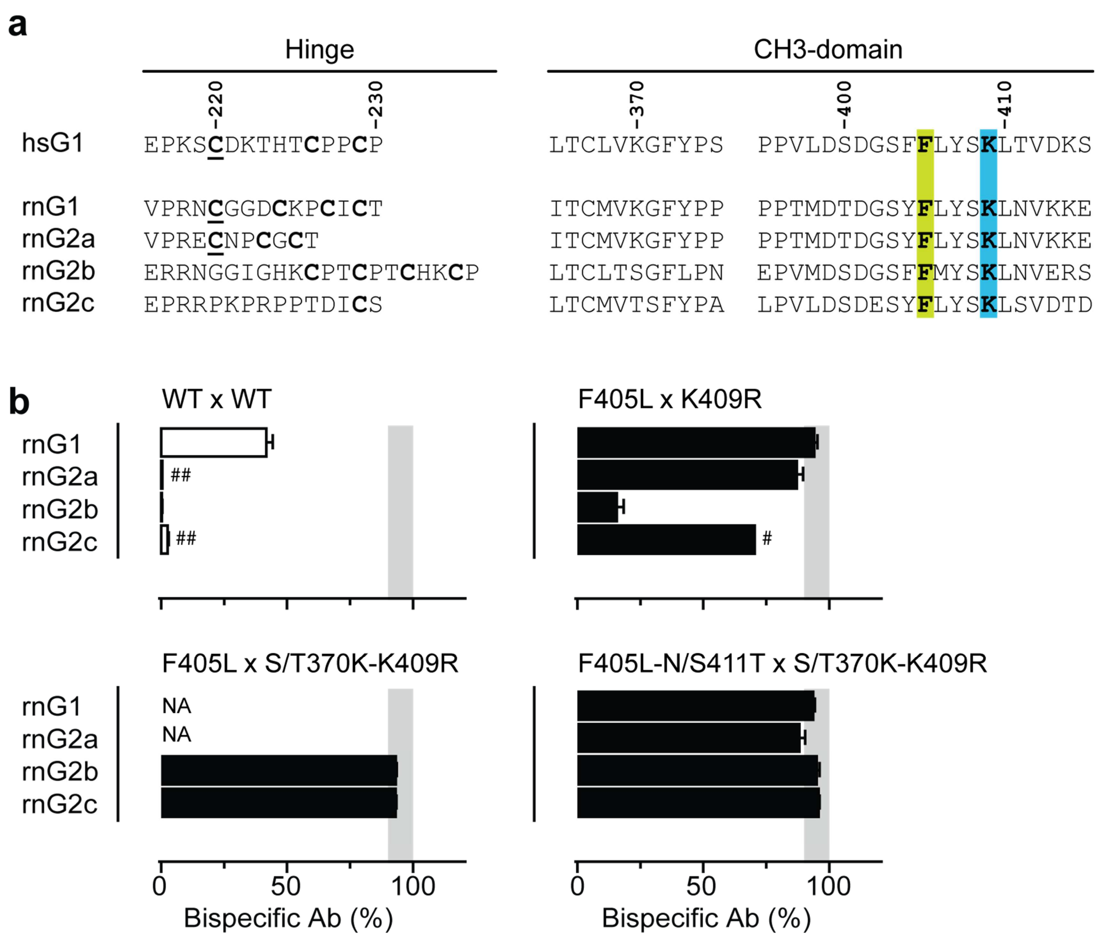

Supplementary Fig. 1. a) Amino acid sequence alignment of human (*H. sapiens*; hs) and rat (*R. norvegicus*; rn) hinge and CH3 regions. EU-numbering convention is used to annotate the amino acid residues. Cysteine residues in the hinge region involved in HC-HC linkage and LC-HC pairing (underlined) are indicated in bold. Residues F405 and K409 are indicated in green and blue, respectively. **b**) Efficiency of cFAE as measured by HIC of mixtures of 2F8-derived and 7D8-derived rnIgG1, rnIgG2a, rnIgG2b or rnIgG2c parental antibodies, containing the indicated mutations (above panels). Data represent mean ± SEM of three separate experiments, except ## = done twice and # =

done once, due to limited sample availability. NA = not applicable. Shaded area represents 90-100% efficiency.

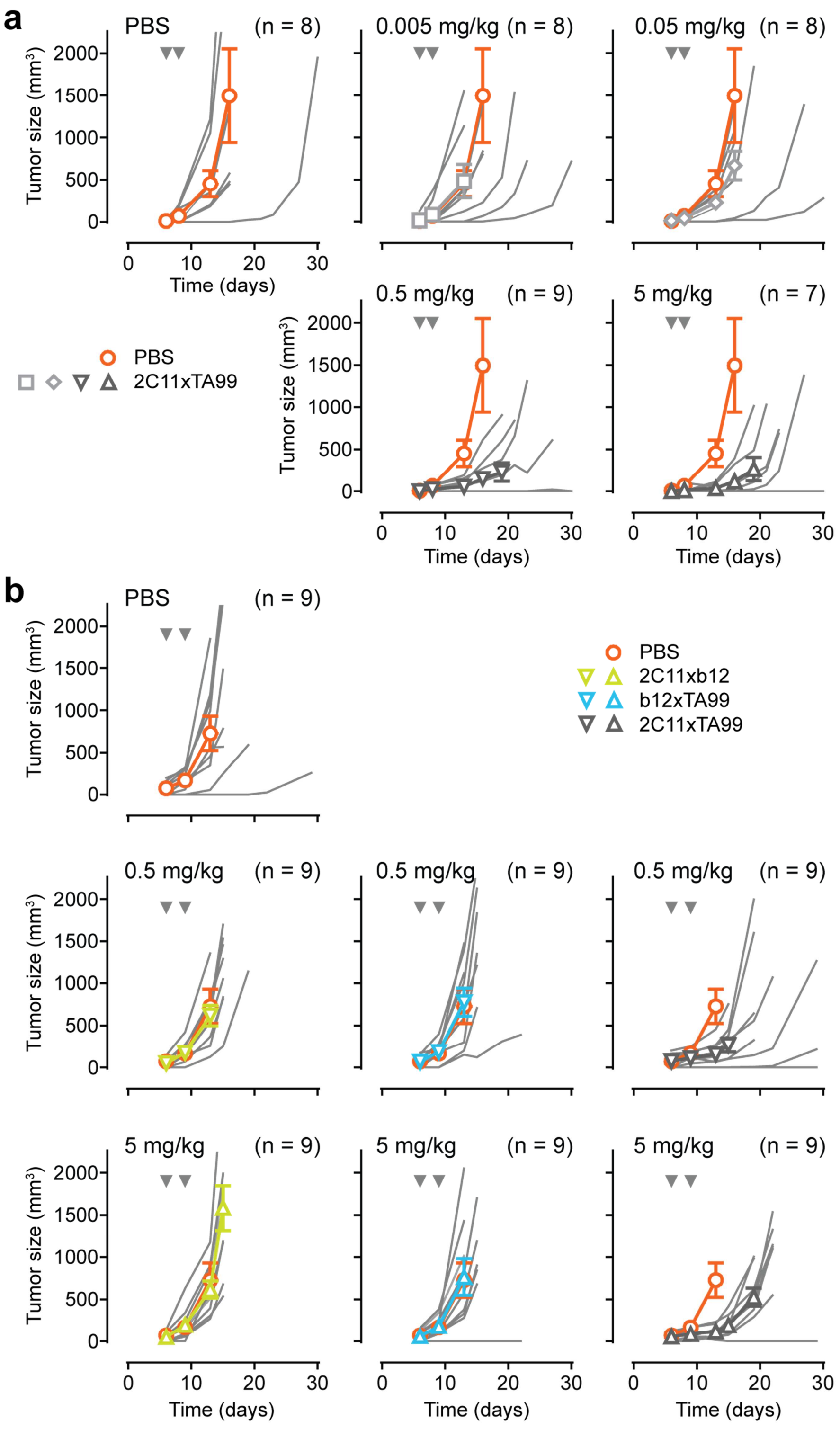

Supplementary Fig. 2. Individual group results of **a**) dose-finding for *in vivo* efficacy of bsIgG2a-2C11xTA99-LALA (data used to generate **Fig 5a**) and **b**)

comparison to functionally monovalent controls (2C11xb12 and b12xTA99) in a syngeneic xenograft model with gp75-expressing B16-F10 tumor cells. On day 6, mice were randomized (n = 7-9 per group) and treated intravenously with the indicated bsIgG2a-2C11xTA99-LALA doses, followed by a second dose at day 8 (**a**) or 9 (**b**) (arrowheads indicate treatment days). The colored symbols connected by thick lines represent mean tumor volumes ± SEM (at time points where groups were still complete). Individual animals are represented by thin grey lines. The PBS control groups (upper left panels) are included as reference in each panel.

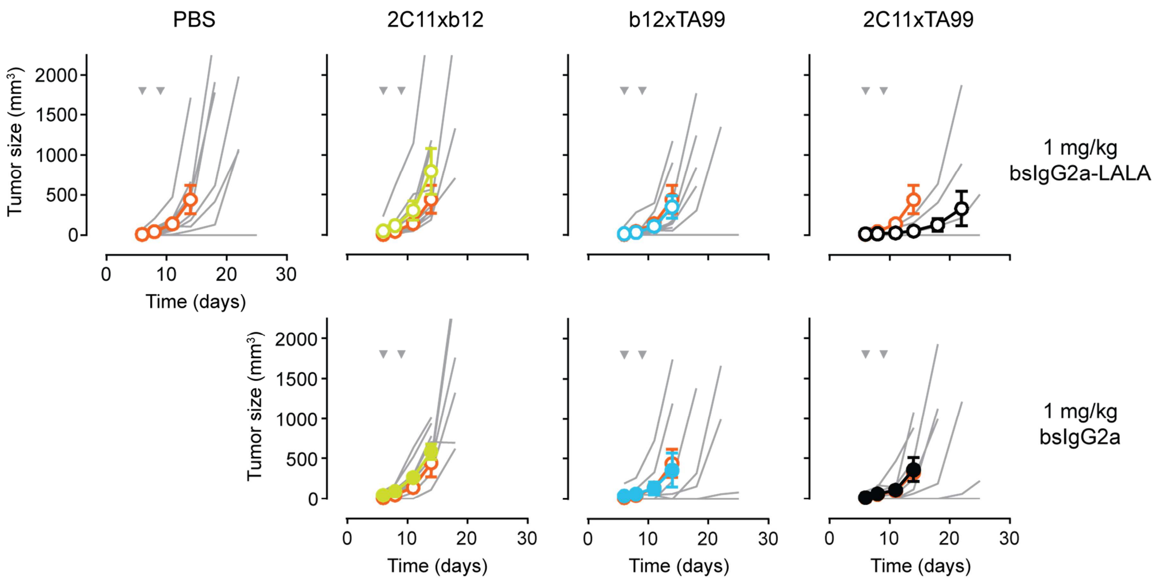

Supplementary Fig. 3. Individual group results of comparison of inert (bsIgG2a-LALA) versus active (bsIgG2a) Fc-backbones on *in vivo* efficacy of 2C11xTA99 bsAbs in a syngeneic xenograft model with gp75-expressing B16-F10 tumor cells (data used to generate **Fig 5b**). On day 6, mice were randomized (n = 9 per group) and treated intravenously with the different bsAb variants (specificities indicated above; backbone indicated to the right) at 1 mg/kg, followed by a second dose at day 9 (arrowheads indicate treatment days). The colored symbols connected by thick lines represent mean tumor volumes ± SEM (at time points where groups were still complete). Individual animals are represented by grey lines. The relevant PBS control groups (upper left panel) is included as reference in each panels.

REFERENCES

1. Teeling, J. L. *et al.* Characterization of new human CD20 monoclonal antibodies with potent cytolytic activity against non-Hodgkin lymphomas. *Blood.* **104,** 1793-1800 (2004).
2. Bleeker, W. K. *et al.* Dual mode of action of a human anti-epidermal growth factor receptor monoclonal antibody for cancer therapy. *J Immunol.* **173,** 4699-4707 (2004).
